# Effect of the topical administration of corticosteroids and tuberculin pre-sensitisation on the diagnosis of tuberculosis in goats

**DOI:** 10.1186/s12917-022-03156-0

**Published:** 2022-01-27

**Authors:** J. Ortega, A. Roy, A. Díaz-Castillo, L. de Juan, B. Romero, J. L. Sáez-Llorente, L. Domínguez, P. Regal, J. A. Infantes-Lorenzo, J. Álvarez, J. Bezos

**Affiliations:** 1 Departamento de Sanidad Animal, Facultad de Veterinaria, Universidad Complutense de Madrid, Madrid, Spain; 2 Centro de Vigilancia Sanitaria Veterinaria VISAVET, Universidad Complutense de Madrid, Madrid, Spain; 3 Ministerio de Agricultura, Pesca y Alimentación, Madrid, Spain; 4 Laboratorio de Higiene, Inspección y Control de Alimentos (LHICA), Departamento de Química Analítica, Nutrición y Bromatología, Facultad de Veterinaria, Universidade de Santiago de Compostela, Lugo, Spain; 5 Unidad de Inmunología Microbiana, Centro Nacional de Microbiología, Instituto de Investigación Carlos III, Majadahonda, Madrid, Spain

**Keywords:** Caprine tuberculosis, Intradermal tests, Corticosteroids, Pre-sensitization, Reactor

## Abstract

**Background:**

Caprine tuberculosis (TB) is a zoonosis caused by members of the *Mycobacterium tuberculosis* complex (MTBC). Caprine TB control and eradication programmes have traditionally been based on intradermal tuberculin tests and slaughterhouse surveillance. However, this strategy has limitations in terms of sensitivity and specificity. Different factors may affect the performance of the TB diagnostic tests used in goats and, subsequently, the detection of TB-infected animals. In the present study, the effect of two of the factors that may affect the performance of the techniques used to diagnose TB in goats, the topical administration of corticosteroids and a recent pre-sensitisation with tuberculin, was analysed.

**Methods:**

The animals (*n* = 151) were distributed into three groups: (1) a group topically treated with corticosteroids 48 h after intradermal tuberculin tests (*n* = 53); (2) a group pre-sensitised with bovine and avian purified protein derivatives (PPDs) 3 days before the intradermal tuberculin test used for TB diagnosis (*n* = 48); and (3) a control group (*n* = 50). All the animals were tested using single and comparative intradermal tuberculin (SIT and CIT, respectively) tests, an interferon-gamma release assay (IGRA) and a P22 ELISA.

**Results:**

The number of SIT test reactors was significantly lower in the group treated with corticosteroids when compared to the pre-sensitised (*p* < 0.001) and control (*p* = 0.036) groups. In contrast, pre-sensitisation with bovine and avian PPDs did not cause a significant reduction in the number of SIT and CIT test reactors compared with the control group. In fact, a higher number of reactors was observed after the prior tuberculin injection in the pre-sensitised group (*p* > 0.05). No significant effect was observed on IGRA and P22 ELISA due to corticosteroids administration. Nevertheless, a previous PPD injection affected the IGRA performance in some groups.

**Conclusions:**

The application of topical corticosteroid 24 h before reading the SIT and CIT tests can reduce the increase in skin fold thickness and subsequently significantly decrease the number of positive reactors. Corticosteroids used can be detected in hair samples. A previous pre-sensitisation with bovine and avian PPDs does not lead to a significant reduction in the number of intradermal tests reactors. These results are valuable in order to improve diagnosis of caprine TB and detect fraudulent activities in the context of eradication programs.

## Background

Animal tuberculosis (TB) is a chronic infectious disease caused principally by *Mycobacterium bovis* and *M. caprae*, the latter being the main cause of TB in goats in Spain [1]. The presence of the disease in goats has a significant impact on the health of both humans and animals, and entails significant economic losses [2–4]. TB eradication programmes in cattle have allowed a significant reduction in the prevalence of the disease and even made it possible for certain countries or regions to attain the officially TB-free status. However, TB in goats is not subjected to compulsory eradication programmes within the EU, while in the case of Spain, certain regions have implemented specific eradication programmes [5]. In this context, diagnosis of TB in goats is included in the new Animal Health Law (Regulation EU 2016/429) with the purpose of movements within the European Union (EU). Moreover, to perform studies that provide data about factors that may affect TB diagnosis will be of paramount importance for TB eradication in goats and others species which can be infected by MTBC members. TB eradication programmes in goats are based principally on test and cull strategies, and the single and comparative intradermal tuberculin (SIT and CIT respectively) tests are the cornerstone of the diagnosis [3].

The SIT/CIT tests have a high specificity at the individual level and an overall high sensitivity at the herd level, but there are certain factors that may affect its performance [6]. The biological potency of the tuberculins used [7], the site of tuberculin injection [8], the period between tests [9], the correct use and maintenance of the injection syringes [10] or the interpretation criteria applied [3] have all been reported as factors that may affect the detection of infected animals when using the SIT and CIT tests. In addition, co-infections with other non-tuberculous mycobacteria or the presence of immunosuppressive diseases or treatments may affect the cell-based immunity on which the official diagnostic tests are based [11].

Furthermore, certain activities may be maliciously carried out in order to alter the results of the TB diagnostic tests. This may be done since the detection of TB in a farm implies culling positive animals (that may have a high value) and restrictions on the movement of animals and the commercialisation of their products, thus having a significant economic impact [12]. These fraudulent activities usually seek to avoid these economic repercussions and are difficult to demonstrate, but they entail large-scale animal and public health problems, along with the consequent damage to the progress of the TB eradication programmes. Among these practices, the topical administration of corticosteroids at the tuberculin inoculation site for fraudulent purposes could potentially be carried out in order to interfere with the intradermal reaction by decreasing the inflammatory process, but no specific studies to assess this risk have been carried out in ruminants. Also, administering tuberculin without respecting the minimum periods between tests (42 days) is known to have an effect on the test results [9], suggesting that it could trigger an anergic status since it prevents the animal’s immune system from recovering properly [13]. This has led to the suspicion that pre-sensitisation with tuberculin prior to SIT/CIT tests could also be also fraudulently used to interfere with the detection of infected animals. However, it is necessary to stress the difficulties involved in demonstrating that these activities have been carried out, which makes it difficult to establish control strategies and corrective measures.

The objective of the present study was to demonstrate whether the topical administration of corticosteroids or a recent pre-sensitisation with tuberculin significantly interferes with the diagnosis of TB when using the SIT and CIT tests. A protocol for the detection of corticosteroids in animal samples was also designed in order to demonstrate the presence of corticosteroid residues at the site of administration.

## Methods

### Experimental design

**Fig. 1:**
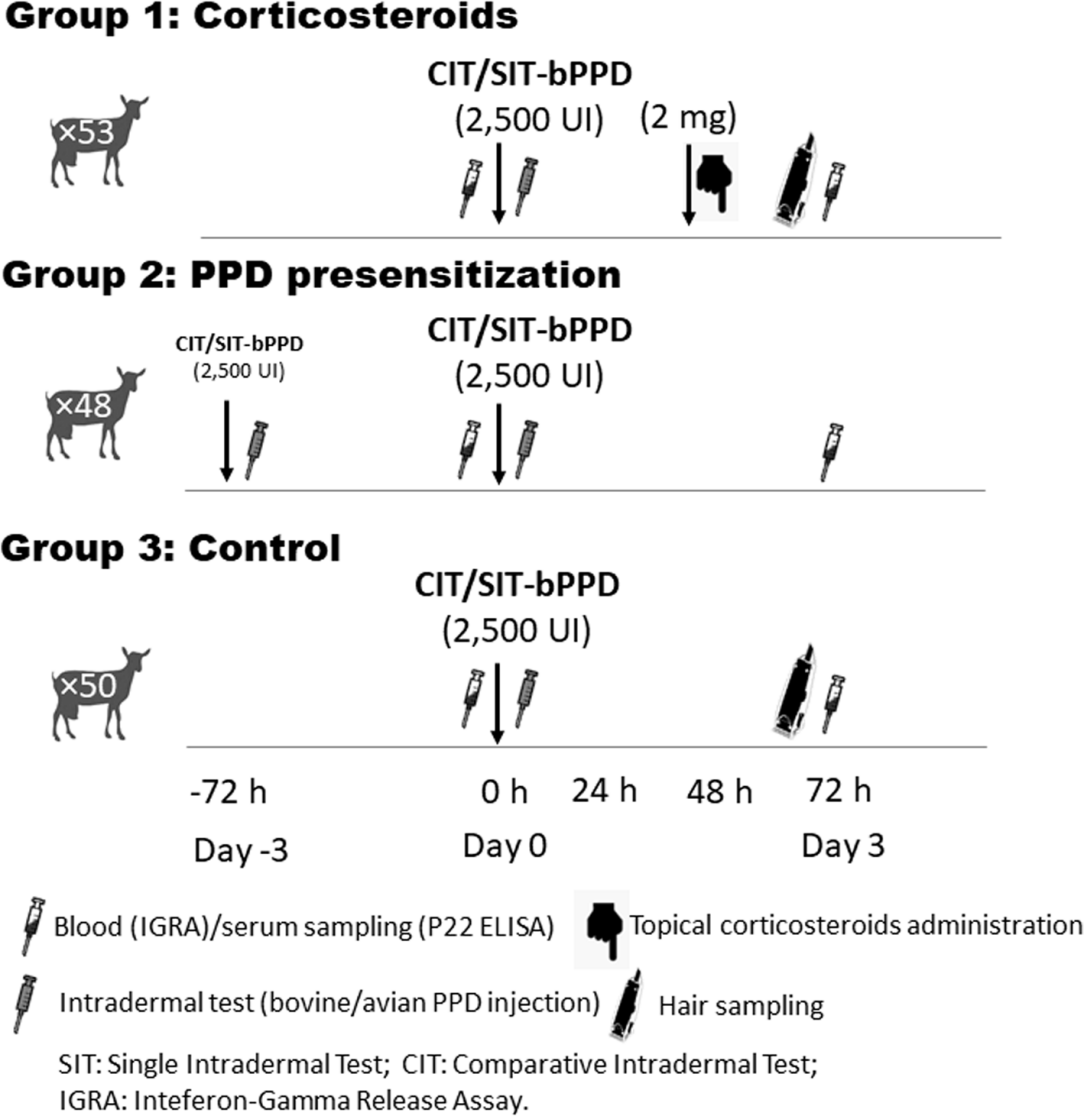
Experimental design

One hundred and fifty-one goats were randomly selected from a *M. bovis-*infected herd (confirmed by bacteriology) with a high apparent prevalence (more than 70% reactors in the previous SIT herd test). The animals were randomly distributed in three experimental groups: (1) treated with corticosteroids (*n* = 53); (2) pre-sensitised with bovine and avian purified protein derivatives (PPDs) (*n* = 48); and (3) control (*n* = 50) (Fig. 1). The pre-sensitised group was subjected to two serial intradermal tests, the first of which was the tuberculin pre-sensitisation (day − 3: first PPD inoculation; day 0: first reading and second PPD inoculation; and day 3: second reading). Simultaneously to the second testing event in the pre-sensitised group, the group treated with corticosteroids (corticosteroids group) and the control group were also subjected to an intradermal test for diagnostic purposes (day 0). In the corticosteroids group, approximately 2 mg of a topical corticosteroid (Betamethasone Valerate 0.5 mg/g, Celecrem, Galenicum Health S.L., Barcelona, Spain) was applied at the inoculation sites (≈ 25 cm^2^ per point of inoculation) 48 h after the SIT/CIT tests (day 2). Blood samples were collected at days 0 and 3 and tested using an interferon-gamma release assay (IGRA) and a P22 ELISA. Finally, hair samples from the inoculation site were collected with a razor blade after the interpretation of the reactions at day 3 (Fig. 1) in order to detect the corticosteroids by means of High Performance Liquid Chromatography-Mass Spectrometry (HPLC-MS).

Animal handling, testing and sampling were performed by qualified veterinarians in accordance with European (86/609/CEE) and Spanish (RD 53/2013) legislation. All procedures were authorised by an institutional ethical committee and approved by the local authorities (PROEX11/18; Comunidad de Madrid). Moreover, the study was carried out in accordance with the Animal Research: Reporting of In Vivo Experiments (ARRIVE) guidelines and a written informed consent to use the animals in the present study was obtained from the owner.

### Intradermal tuberculin tests

The intradermal inoculations of 0.1 ml of avian and bovine protein purified derivatives (PPD-A and PPD-B; CZ Vaccines, Porriño, Spain) were carried out at day − 3 and day 0 in the pre-sensitised group and only at day 0 in the control and corticosteroid groups (Fig. 1). The avian and bovine PPDs (2500 UI/0.1 ml) were injected on the right-medial and left-medial side of the neck, respectively using a Dermojet syringe (Akra Dermojet, Pau, France), and the reactions were interpreted 72 h later (day 3) by the same veterinarian, as described previously [14]. The SIT and CIT tests were performed in accordance with the protocol published by the European Union Reference Laboratory (EU-RL) for bovine TB following Regulation EU 2016/429, Commission Delegated Regulation EU 2020/688 and Royal Decree RD2611/1996. An increase in the skin fold thickness (SFT) of ≥4 mm or the presence of clinical signs (exudation, oedema or necrosis) were considered a positive reaction to the SIT test. Animals were considered positive to the CIT test if they had a positive bovine reaction that was more than 4 mm greater than the avian reaction or they showed clinical signs at the bovine PPD inoculation site.

### Interferon-gamma release assay (IGRA)

Whole blood samples were collected from animals in the three groups in lithium heparin at day 0 and day 3 for the detection of IFN-γ production (Table 1). These blood samples were then stimulated with bovine and avian PPDs (CZ Vaccines, Porriño, Spain) at a final concentration of 20 μg PPD/mL, as described previously [15]. A control aliquot of each sample was stimulated with phosphate buffered saline (PBS). The blood was incubated for 18–20 h at 37 °C in a humidified atmosphere. The samples were then centrifuged for 15 min at 770 g and the supernatant was collected. The IFN-γ release in plasma was measured using a commercial IGRA (Bovigam TB kit, Thermo Fisher Scientific, Waltham, USA) according to the manufacturer’s instructions. A reaction was considered to be positive when the optical density (OD) of a sample stimulated with bovine PPD minus the OD of PBS was greater than or equal to 0.05 and greater than the OD of the sample stimulated with avian PPD.

**Table 1:** Number of positive reactors (and percentage with Wilson’s 95% confidence intervals) to the different diagnostic tests used in each experimental group

Day 0 (N, % and 95 CI)					Day 2 (N, % and 95 CI)		Day 3 (N, % and 95 CI)			
Group	SIT test	CIT Test	IGRA	P22 ELISA	SIT Test	CIT test	SIT test	CIT test	IGRA	P22 ELISA
Corticosteroids (*n* = 53)	NA	NA	22, 41.5% (29.3–54.9)	21, 39.6% (27.6–53.1)	34, 64.2% (50.7–75.7)	10, 18.9% (10.6–31.4)	23, 43.4% (31–56.7)	10, 18.9% (10.6–31.4)	35, 66% (52.6–77.3)	23, 43.4% (30.9–56.7)
Pre-sensitized (*n* = 48)	39, 81.3% (68.1–89.8)	22, 45.8% (32.6–59.7)	37, 77.1% (63.5–86.7)	25, 52.1% (38.3–65.5)	NA	NA	41^a^, 85.4% (72.8–92.8)	22^a^, 45.8% (32.6–59.7)	31, 65.6% (50.4–76.6)	25, 52.1% (38.3–65.5)
Control (*n* = 50)	NA	NA	28, 56% (42.3–68.8)	17, 34% (22.4–47.9)	NA	NA	32, 64% (50.1–75.9)	13, 26% (15.9–39.6)	36, 72% (58.3–82.5)	21, 42% (29.4–55.7)

*SIT* Single Intradermal Test, *CIT* Comparative intradermal test, *IGRA* Inteferon-Gamma Release Assay, *NA* Not applicable; ^a^With regard to the skin fold thickness measured at day −3

### P22 ELISA

An in-house indirect enzyme-linked immunosorbent assay based on the multiprotein complex P22 (P22 ELISA) for the detection of specific antibodies against the *Mycobacterium tuberculosis* complex (MTBC), obtained from the inmunopurification of the bovine PPD (CZ Vaccines, Porriño, Spain) by affinity chromatography [16], was performed on samples collected at day 0 and day 3. The ELISA was carried out as described previously [17]. Briefly, the plates were coated with p22 at 10 μg/ml and then incubated overnight at 4 °C. Following one wash with PBS solution containing 0.05% Tween 20 (PBST), wells were blocked with 5% skim milk powder solution in PBS during 60 min at Room Temperature (RT). Serum samples were added in duplicate at 1:100 dilution in skimmed milk and incubated for 60 min at 37 °C and subsequently washed with PBST three times. After, one-hundred microliters of horseradish peroxidase (HRP)-conjugated rabbit anti-sheep IgG antibodies (SouthernBiotech, Birmingham, USA) were added and the plates were incubated for 30 min at RT. Following 5 washes with PBST, colour was developed by adding 100 μl of o-phenylenediamine dihydrochloride substrate (FAST OPD, Sigma–Aldrich, St Louise, USA) incubated for 6 min in darkness and RT conditions. Then, the reaction was stopped with 50 μl of H2SO4 (3 N) and the OD were measured at 492 nm with an ELISA reader. The negative controls of each plate were considered as the internal control of the plate and the OD of the negative control must be less than 0.2. The sample results were expressed as an ELISA percentage (E%), which was calculated by using the following formula: [sample E% = (mean sample OD/2 × mean of negative control OD) × 100]. The cut-off value was defined as the ratio of the mean sample OD to the double of mean OD of the negative control. Therefore, the cut-off of each plate was based on the OD of the negative controls belonged to each plate. Serum samples with E% values greater than 150 were considered to be positive.

### High performance liquid chromatography-mass spectrometry (HPLC-MS)

All solvents were of HPLC-MS or analytical grade and were supplied by Merck (Madrid, Spain). Betamethasone 17-valerate (BMV) was obtained from Sigma-Aldrich (Madrid, Spain). The acetate buffer solution was prepared using sodium acetate anhydrous (Merck, Madrid, Spain) at 1 M and adjusting the final pH to 4.8 using acetic acid. A stock solution of BMV was prepared in methanol at a concentration of 100 μg mL-1 and preserved at − 20 °C.

Each disposable razor blade containing a hair sample was separated from its handle and placed in a 50 mL conical tube, after which 10 mL of acetate buffer solution (1 M, pH 4.8) were added. The tubes were capped, vortexed for 1 min and sonicated in an ultrasonic bath for 30 min in order to facilitate the release of all the hair trapped in the razor blades. The samples were then placed on a rocker table, where they were left to shake continuously overnight at 4 °C. After removing the blades from the tubes with clean tweezers, 10 mL of tert-butyl methyl ether were added and the samples were once again placed on the rocker table to shake continuously for 120 min at 4 °C. They were subsequently centrifuged at 2500 rpm for 15 min, and 1 mL of tert-butyl methyl ether layer was placed in a glass tube and evaporated under a nitrogen stream at 37 °C. The dried samples were dissolved in 1 mL of water with 0.1% of formic acid, and 10 μl were injected into the HPLC-MS/MS system in order to determine BMV. One calibration curve was prepared in acetate buffer solution and submitted to the extraction protocol on each day of analysis. Calibrators were used for the quantification of BMV using the peak area.

The samples were analysed by employing LC-MS/MS. The HPLC system consisted of a quaternary pump, a degasser, a column oven and an 1100 series auto-sampler (Agilent Technologies, Minnesota, USA). A Phenomenex Synergi 2.5 μm MAX-RP 100A (100 × 2 mm) column and guard column (Torrance, CA, USA) were used for analyte separation at 30 °C. The mobile phase was acetonitrile mixed on a gradient mode with water with 0.2% of formic acid, at a flow rate of 300 μl min–1.

A Q-Trap 2000 mass spectrometer with an Ion Source Turbo Spray (Applied Biosystems MSD Sciex, Toronto, Canada) was then used, working in ESI positive mode. The MRM transitions monitored for betamethasone 17-valerate were m/z 477 > 355 (quantification) and 477 > 337 (qualification). Nitrogen was produced by a high purity nitrogen generator (PEAK Scientific Instruments, Chicago IL) and used as a curtain, nebulizer and collision gas. Data was collected using a Dell Optiplex GX400 workstation and processed by employing an Analyst 1.4.1 software package (MDS SCIEX).

### Statistical analysis

Wilson’s 95% confidence intervals (95% CI) were calculated for the percentage of reactors to the different techniques using WinPepi, version 11.6 [18]. The proportions of test reactors in each group were compared using the chi-square test, while the proportions of test reactors within each group at days 0, 2 (only corticosteroid group) and 3 were compared using McNemar’s test. Quantitative values, such as the increase in the SFT, IFN-γ levels (OD) and the ELISA result (E%) of animals in the different groups were compared using the Kruskal-Wallis test followed by pairwise tests for multiple comparisons of mean rank sums after the adjustment of the *p* value using the Bonferroni correction. Potential differences in the increase in SFT, the OD and the differences in E% for measures from animals in a given group at days 0, 2 (only corticosteroid group) and 3 were analysed by means of the Wilcoxon signed-rank test. All the analyses were carried out using SPSS Statistics 25 (IBM, New York, NY, USA), and interpreted by considering a *p*-value of 0.05 in order to determine statistical significance.

## Results

### Cell-based diagnostic tests

**Fig. 2:**
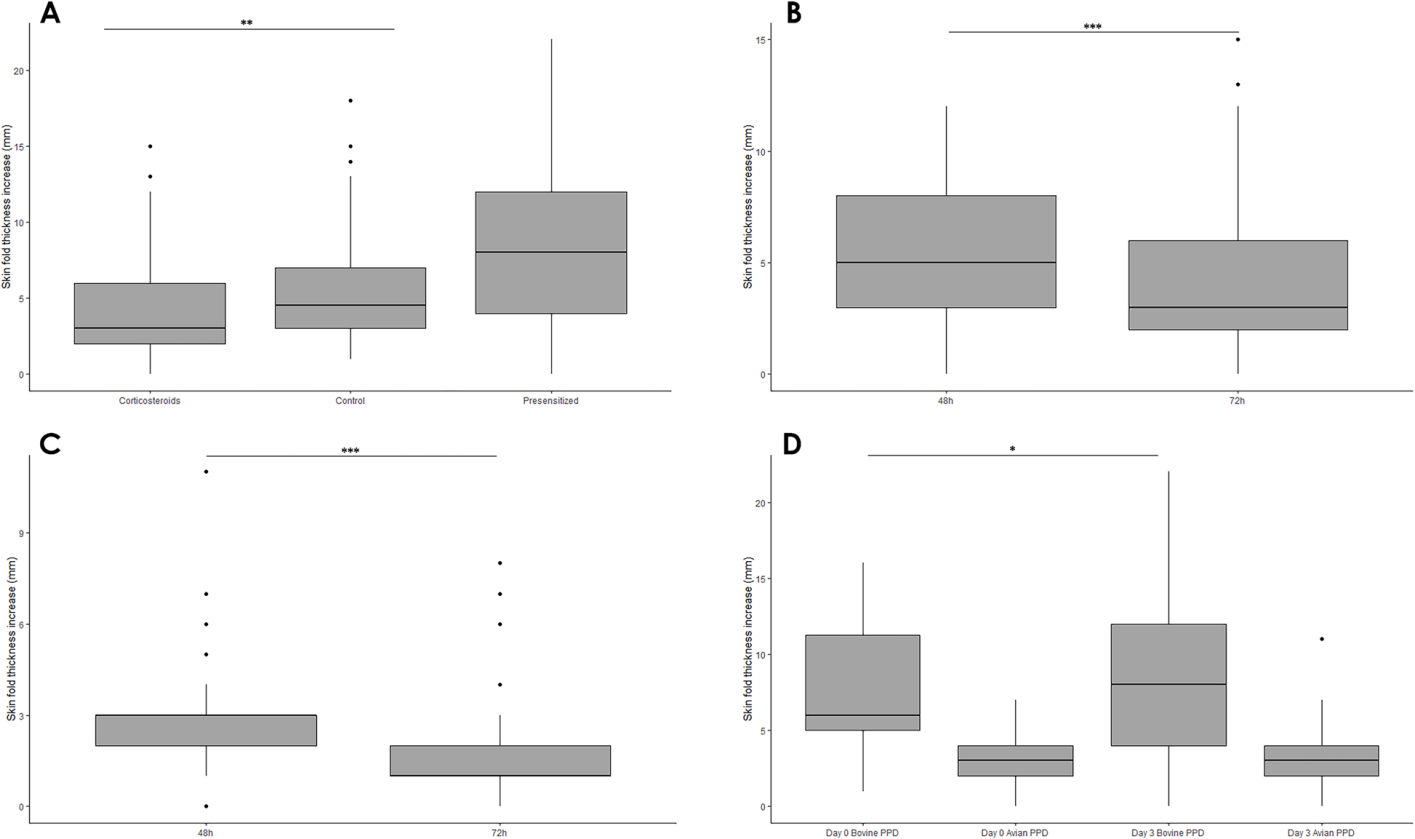
Summary of the differences in median skinfold thickness (mm) after bovine PPD injection in the corticosteroid, control and pre-sensitised groups at day 3 (**A**), after bovine (**B**) and avian (**C**) PPD injection in the corticosteroid group at 48 h and 72 h, and after bovine and avian PPD injection in the pre-sensitised group at days 0 and 3 (**D**). The frequency of skinfold thickness measures observed in each group is correlated with the box of the figure whose medians are represented as the black lines. Significant differences are described in the boxplot as follows: *** *p* < 0.001, **p = 0.01, *p < 0.1

The number and percentage of positive reactors to the different diagnostic tests are shown in Table 1. The number of SIT test reactors was significantly lower in the corticosteroid group when compared to the control (*p* = 0.036) and pre-sensitised (*p* < 0.001) groups at day 3. This was also reflected by a lower increase in the SFT at day 3 in the corticosteroid group when compared with the control group (*p* = 0.010, Fig. 2A). The median increase in SFT at the bovine PPD injection site was below the cut-off value when using the standard interpretation of the SIT test for the corticosteroid group (Me = 3 mm, IQR 2–6.5), and was 1.5 mm lower than the median value observed for the control group (Me = 4.5 mm, IQR 3–7.25). Furthermore, the effect of the topical administration of corticosteroids on the SIT test was confirmed by a significant decrease in the SFT at the PPD bovine inoculation site between day 2 (48 h, time of application) and day 3 (72 h, time of SIT test interpretation) (*p* < 0.001, Fig. 2B). A similar pattern was observed with regards to the increase in SFT at the avian PPD inoculation site (*p* < 0.001, Fig. 2C), although in this case, the qualitative results (overall number of reactors to the CIT test) did not differ between 48 and 72 h (Table 1).

A previous pre-sensitisation with bovine and avian PPDs (72 h before the SIT test used for diagnosis) did not lead to a significant reduction in the number of reactors between day 0 and day 3 in the pre-sensitised, and the increase in the median SFT difference observed between day 0 and day 3 was not significant (*p* = 0.097) (Fig. 2D). In fact, the number of reactors increased in comparison to the results observed for the previous test (when PPD was injected for pre-sensitisation), and a significantly higher number of reactors were observed when compared to the animals in the control group at day 3 (*p* = 0.015).

The topical application of corticosteroids had no effect on the IGRA, since there were no significant differences between the corticosteroid group and the control group as regards the number of reactors (*p* = 0.513). Moreover, there were no significant differences between the groups either on the quantitative IFN-γ result (OD) at day 3 (*p* = 0.340). The number of IGRA positive animals observed in the pre-sensitised group when compared to the control group was significantly higher at day 0 (*p* < 0.01). In this sense, a significant increase in the IFN-γ levels was observed between day 0 and day 3 in the control (*p* = 0.001) and corticosteroid (*p* < 0.001) groups. However, a significant decrease on the quantitative IFN-γ result was observed in the pre-sensitized group between day 0 and day 3 (*p* = 0.007). As expected, a higher number of reactors was observed in the control (*p* = 0.057) and corticosteroid (*p* = 0.004) groups in the IGRA performed at day 3 compared to day 0 (Table 1). In contrast, a lower number of reactors in the pre-sensitized group was detected in the IGRA performed at the second intradermal test although the differences were not significant (*p* = 0.307) (Table 1).

### Antibody-based diagnostic test (P22 ELISA)

A significant increase in the E% was observed between day 0 and day 3 for the corticosteroid (*p* < 0.001), pre-sensitised (*p* = 0.002) and control (*p* = 0.001) groups. Nevertheless, the differences between the groups at day 3 in the E% were not significant (*p* = 0.248). Additionally, the differences between the groups in terms of the number of positive animals at day 3 were not significant (*p* = 0.555).

### Corticosteroids detection

The HPLC-MS detected residues of Betamethasone Valerate in all the hair samples collected at the site of administration except one (*n* = 52/53, 98.1%). This goat was a positive reactor to the SIT and CIT tests and its SFT decreased by 3 mm between the time of application and the skin test interpretation at day 3. The amount of analyte detected in the hair samples collected using disposable razor blades ranged from 1 to 9 μg/sample (Me = 3 μg, IQR 1.5–5).

## Discussion

In the present study, the topical administration of corticosteroids at the intradermal injection site 24 h before reading had a significant effect on the results of the SIT/CIT tests carried out on TB infected goats. In contrast, the use of topical corticosteroids had no significant effect on the IGRA and P22 ELISA results. Moreover, pre-sensitisation with tuberculin 72 h before the SIT/CIT tests did not reduce the reactivity to intradermal test.

The administration of corticosteroid at the PPD injection site 24 h before interpreting the SIT/CIT tests reduced the difference in SFT when compared to the control group, thus leading to false negative results. We observed a significant decrease in the number of positive reactors to the SIT and CIT tests in goats. Previous studies have evaluated the effect of different corticosteroids and non-steroid anti-inflammatory substances on the immune response in cattle [19–22]. Maślanka and collaborators observed that dexamethasone significantly reduced the percentage of IFN-γ producing cells in CD25-CD4+ and CD25-CD8+ lymphocytes in cattle, unlike meloxicam or flunixin meglumine [20–22]. Goff associated the treatment with dexamethasone with suppressed PPD-stimulated IFN-γ production, which may be interpreted as false negative results [19]. However, to the authors’ knowledge, this is the first study to evaluate the effect of corticosteroids on SIT/CIT tests and other TB diagnostic techniques in ruminants. We have demonstrated the ability of betamethasone to interfere with the TB diagnosis by decreasing the number of reactors to SIT/CIT tests and, have therefore, shown its potential usefulness for fraudulent purposes. Further studies are required in order to investigate the effect of other corticosteroids, other substances such as non-steroid anti-inflammatory drugs or antibiotics, different administration routes and times of application on the official TB diagnostic techniques, and to develop efficient methods to detect them in order to prevent potentially frauds.

The reduction of reactivity in *M. bovis* infected animals after a recent administration of PPDs has also been reported in several studies on cattle [13, 23–25]. Previous studies reported a period of desensitisation after PPD inoculation, during which the response to consecutive intradermal tests was reduced by 8 weeks or more [13, 25, 26]. Other studies have reported this phenomenon using high doses of tuberculin administered by routes other than those routinely used [23] or in cattle previously sensitised with *M. bovis* [24]. However, to the authors’ knowledge, there are no previous studies in scientific literature evaluating the effect of repeated tuberculin skin testing in goats. In our study, the pre-sensitisation with tuberculin 72 h before the SIT/CIT tests did not significantly affect the test results obtained for goats. Previous studies in bovines, in which animals were subjected to two successive skin tests with a short interval of 4 or 7 days between tests, reported a significant skin-test desensitisation in reactor cattle [24, 27]. It is necessary to stress that, in general, the interval between two official skin tests is, in the context of the regional eradication programmes, of several months-1 year for TB-free herds and at least forty-two days for TB-infected herds. According to our results, a recent pre-sensitisation with tuberculin in goats would not reduce reactivity to the SIT/CIT tests.

With regard to the effect that the topical administration of corticosteroids had on the IGRA results animals treated with corticosteroids, a previous study showed that the application of parenteral dexamethasone reduced IFN-γ production in TB-infected cattle [19]. Furthermore, other similar corticoid formulations, such as dexamethasone, have been associated with a decrease in IGRA values as the result of a suppression of the lymphocyte function, which may entail false negative results [19, 28–30]. It has also been demonstrated that dexamethasone significantly reduces the percentage of IFN-γ producing cells in the CD25^−^CD4+ and CD25^−^CD8+ lymphocytes in cattle, unlike other anti-inflammatory substances such as meloxicam [21, 22]. However, under the conditions of our study, the topical administration of corticosteroids did not have a systemic anti-inflammatory effect and did not reduce the number of positive reactors to IGRA, since the differences in the number of reactors observed in the corticosteroid group and the control group were not significant, probably due to the topical administration and doses used. In fact, an increase of the quantitative values (OD) was observed in this group, probably associated to a booster effect due to the previous tuberculin administration, since the same effect was observed in the control group. In this respect, further studies are required in order to investigate the possible effect on IGRA results after systemic corticoid treatment in goats.

In the present study, in spite of the decrease of the IFN-γ levels observed in this group, the number of IGRA positive animals was also unaffected by the recent pre-sensitisation with tuberculin. These results are in agreement with previous studies in TB-infected cattle that demonstrated that the CIT test did not have a significant effect on IGRA results when blood was collected 3–10 days after the intradermal test [9, 13, 31–33]. It has, however, been reported that the caudal fold test, which is the official test used on bovines in New Zealand and the United States [34, 35], can increase the IFN-γ release 3 days after the skin test [33, 36, 37]. Moreover, different effects of the CIT and caudal fold tests on the IGRA reactivity were observed in a previous study [13]. Coad and collaborators reported that IFN-γ levels increased in animals naturally infected with *M. bovis* and subjected to the caudal fold test, whereas no effect was observed after the CIT test [13]. Moreover, a recent study reported that a prior exposure to *M. avium* or environmental mycobacteria induced a significant increase in the IFN-γ response after CIT tests in cattle [38].

A significant increase in the antibody response (E% using P22 ELISA) was observed in the corticosteroid group 72 h after PPD inoculation. This booster effect has been widely described in ruminants and can be used to increase the sensitivity of the skin test [39–43]. The antibody titres and the sensitivity of the serial use of serological tests are maximized between 15 or 30 days after the skin test [39, 41, 44]. However, the increase in antibody levels can be detected just 3 days after PPD inoculation in goats [44], as we observed. In our study, the topical administration of betamethasone did not have a significant effect on the humoral immune response in goats. In this context, a previous study showed that corticosteroids did not cause a selective depletion of B-lymphocytes, some of which differentiate into plasma cells and produce antibodies in cattle [45]. In the present study, the differences between the number of positives to P22 ELISA in the corticosteroid group and the control group were not significant, probably because of the topical administration to which these animals were subjected. With regard to the pre-sensitised animals, we observed a significant increase in the E% 3 days after the skin tests when compared to day 0. However, there were no differences in the number of positive animals between day 0 and day 3. Moreover, there were no significant differences between groups as regards the number of animals that tested positive to the P22 ELISA at day 3, thus signifying that pre-sensitisation with tuberculin and the topical administration of betamethasone had no effect on the humoral response.

Various analytical methods can be employed to detect corticosteroids and other substances that can interfere with TB diagnostic tests. The development of effective protocols and techniques to detect these substances when there is a suspicion of fraud is of paramount importance. The present study demonstrates that corticosteroids can be detected by HPLC technique on hair samples collected from the PPD inoculation site, and that it can be a valuable tool to confirm its presence in the case of suspected topical administration in goats. However, further studies are required in order to determine whether hair samples are the most appropriate specimen to detect corticosteroids systemically administered to ruminants, or whether other samples such as serum would be more suitable.

## Conclusions

In conclusion, the results obtained in our study have made it possible to characterise the effect of pre-sensitisation with tuberculin and the topical administration of corticosteroids on the SIT and CIT tests, IGRA, and P22 ELISA results in goats, activities that could be used in domestic ruminants in the context of TB eradication programmes to affect the results of diagnostic tests. Our study demonstrates that corticosteroids can interfere with the SIT/CIT test results in goats, whereas a recent previous PPD inoculation does not significantly affect the results of the tests. Our study also describes an efficient method to investigate the presence of topical corticosteroids, thus contributing to the detection of activities with fraudulent purposes which seriously impair the satisfactory progress of the TB eradication programmes.

## Data Availability

The datasets used and/or analysed during the present study are available from the corresponding author on reasonable request.

## Abbreviations

BMV: Betamethasone 17-valerate
95% CI: Wilson’s 95% confidence intervals
CIT: Comparative intradermal tuberculin
E%: Elisa percentage
EU-RL: European Union Reference Laboratory
HPLC-MS: High performance liquid chromatography-Mass Spectrometry
IGRA: Interferon-gamma release assay
MTBC: *Mycobacterium tuberculosis* complex
OD: Optical density
PBS: Phosphate buffered saline
PPD-A: Avian protein purified derivative
PPD-B: Bovine protein purified derivative
SFT: Skin fold thickness
SIT: Single intradermal tuberculin
TB: Tuberculosis
